# Sickle cell leg ulcer successfully managed by hyperbaric oxygen: a case report

**DOI:** 10.3389/fmed.2023.1171971

**Published:** 2023-06-15

**Authors:** Awni Alshurafa, Mohammed Alkhatib, Mohammad Abu-Tineh, Mohamed A. Yassin

**Affiliations:** ^1^Hematology Department, Hamad Medical Corporation, Doha, Qatar; ^2^Internal Medicine Department, Hamad Medical Corporation, Doha, Qatar

**Keywords:** sickle cell disease, leg ulcer, hyperbaric oxygen, wound care, wound healing

## Abstract

Sickle cell leg ulcers (SCLUs) are usually chronic, painful, and devastating complications of sickle cell disease. Skin vaso-occlusion with compromised blood flow, chronic inflammation, and endothelial dysfunction is thought to be the underlying mechanism. It is usually slow to heal, and it may become chronic and superinfected. The management of SCLUs is usually challenging and requires a multidisciplinary team. Multiple systemic and local therapies have been tried in SCLU treatment. However, the outcome is variable: currently, there are no official recommendations for the best effective treatment. Herein, we report a 34-year-old male patient with non-transfusion-dependent sickle cell disease who was suffering from a chronic left ankle ulcer and was successfully managed with hyperbaric oxygen therapy, resulting in a complete resolution of this devastating complication.

## Introduction

Sickle cell disease (SCD) is an inherited disorder that results from a point mutation in the beta-globin gene leading to the production of sickle hemoglobin. When the red blood cells become deoxygenated, it leads to abnormal hemoglobin S polymers that destroy the normal shape of the red cell. These rigid sickled-shaped red blood cells can subsequently occlude blood flow in many parts, leading to both acute and chronic tissue damage (1–3).

Sickle cell leg ulcers (SCLUs) are relatively common and can be disabling complications of sickle cell disease. The pathogenesis is complex and may include mechanical obstruction, bacterial infection, local thrombi, and deficiency of functional nitric oxide leading to endothelial dysfunction. The geographic origin is an important factor in the occurrence of SCLUs, with a reported prevalence of 40% in tropical areas like Jamaica compared to approximately 18% in the USA (4, 5).

Multiple systemic and local therapies have been tried in SCLU treatment. However, the outcome is variable: currently, there are no official recommendations for the best effective treatment (6, 7). Hyperbaric oxygen is a treatment modality for different medical conditions. Accelerated wound healing may be mediated by the combined effects of reactive oxygen species and the increased production of nitric oxide, an essential cellular signal for tissue repair (8, 9).

Herein, we report a 34-year-old male patient with sickle cell disease who was suffering from chronic recurrent left ankle ulcers and received four sessions of hyperbaric oxygen therapy, which resulted in the complete resolution of his chronic ulcers.

## Case presentation

A 33-year-old gentleman presented with a known case of sickle cell disease and was on folic acid and analgesics as needed. With regular follow-ups in the hematology clinic, the hematological laboratory values range for hemoglobin was between 8 and 9 gm/dl without transfusions, platelets were between 200 and 300 **×** 10^9^/L, hemoglobin S was 91.4%, hemoglobin F was 3.9%, total bilirubin was 32 μmol/L, indirect bilirubin was 21 μmol/L, LDH was 503 u/l, and retic percentage was 7.9%, and his disease was under control without hydroxyurea. Throughout the course, he did not show any clinical manifestations of SCD, such as stroke, retinopathy, acute chest syndrome, and priapism. Moreover, he was physically active without any limitations, and the latest painful crisis was 4 years ago. For the last 6 months, he was suffering from a left ankle ulcer on the left medial and lateral malleoli for which he was following up in the podiatry clinic for regular dressings.

Despite that, after 2 months, the ulcers continued to expand and became more painful, so cultures were sent and the results were negative. An MRI of the ankle was performed, which showed an increased signal with irregular outline synovium of the ankle joint due to synovitis and the posterior lateral part of the calcaneum adjacent skin thickening and subcutaneous edema extending distally to the level of the calcaneocuboid articulation and to a lesser extent adjacent to its medial part.

**Figure 1:**
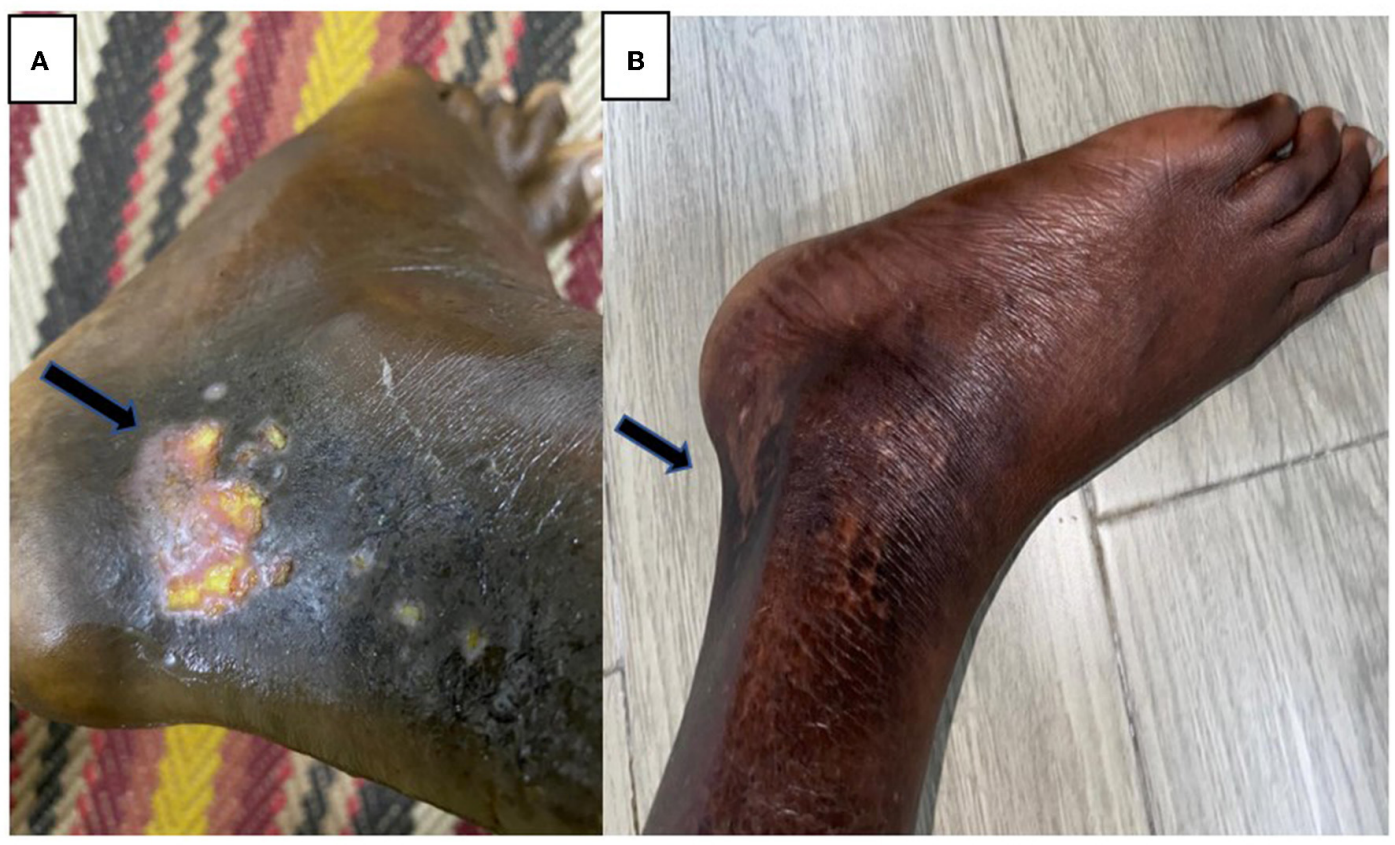
**(A)** Left ankle ulcer before using HBOT. **(B)** Almost complete resolution of his ulcers after using HBOT.

After that, in the podiatry clinic, they tried different types of dressing, and then he was referred to orthopedic and pain clinics with minimal resolution of his ankle ulcers, which were disabling and devastating for him (Figure 1A).

After 4 months, the ulcers increased in size with a length of 1.9 cm and width of 1.5 cm and became extremely painful, so cultures were sent from the wound, which did not grow any organisms, and subsequently, conservative measures with regular dressings, leg elevation, offloading, physiotherapy, and pain medications were tried but without significant clinical improvement.

Consequently, we decided to change our management strategy. Therefore, we counseled our patient about hyperbaric oxygen therapy to treat the ankle ulcer and reduce pain, and he agreed.

He received four sessions of hyperbaric oxygen therapy in 4 weeks, with one session per week that lasted for 2 h with a pressure of 2.5 pounds per square inch. Afterward, he achieved dramatic clinical improvement (without blood transfusion), and the examination showed well-granulated wounds. He had less pain and was healing properly without signs of active infection. He tolerated HOBT sessions very well without notable complications. Subsequent follow-up showed almost complete resolution of his ulcers (Figure 1B), and he was able to practice his daily life activities without limitations.

## Discussion

Sickle cell leg ulcersare associated with significant medical and psychological morbidity and are an independent risk factor for death. They may occur spontaneously or after trauma with medial and lateral malleoli being the most common sites. They are usually slow to heal and may become chronic and superinfected. Risk factors for chronicity include venous incompetence, lower socioeconomic status, older age, a high degree of hemolysis, geographic distribution, and low steady-state hemoglobin (5, 10, 11).

Prevention is the best approach to sickle cell leg ulcer management, including avoiding trauma, wearing properly adapted shoes, preventing insect bites, and early counseling in case of skin injury. One study showed that a hemoglobin F of more than 25% is needed in order to reduce leg ulcer incidence by one-third (12). However, when a sickle cell disease patient develops a leg ulcer, the mainstays of treatment are non-specific measures with local wound care, pain control, bedrest, treatment of infection, venous compression therapy, and general sickle cell disease-based therapy chronic blood transfusion and hydroxyurea. Moreover, there are multiple treatment options that may be of benefit but remain unproven due to the lack of sufficient evidence. Options include Apligraf (a skin equivalent), hyperbaric oxygen, RGD peptide matrix, topical timolol, topical sodium nitrite cream, topical triple anti-biotherapy, solcoseryl, and collagen/glycosaminoglycan matrix (7, 13). The role of novel sickle cell disease drugs in the management of leg ulcers is not yet clear (14–16).

Hyperbaric oxygen (HBO) is a treatment modality for various conditions. It works by supplying oxygen at a pressure greater than the sea level leading to an increase in oxygen levels in the blood and body tissues. HBO has been used in chronic wounds that have not improved with conventional measures. This includes surgical and traumatic wounds, diabetic foot ulcers, skin grafts, and reconstructive flaps with vascular compromise. The use of HBO has not been specifically studied in sickle cell leg ulcer management. A recent meta-analysis evaluating the role of hyperbaric oxygen therapy in chronic wound healing showed that diabetic foot ulcers significantly improved in the short-term follow-up; however, this benefit was not demonstrated in the long term. However, it did not show any benefits of hyperbaric oxygen therapy in other chronic wounds. Sickle cell leg ulcer was not specifically studied. In our reported case, the patient was suffering from a painful chronic ulcer that did not improve with usual conservative measures, including regular dressing with different creams, leg elevations, pain management, and antibiotics. Using hyperbaric oxygen relieved his pain and resulted in almost complete healing. This finding supports the need for further large studies to confirm this therapeutic benefit (17, 18).

Hyperbaric oxygen improves wound healing by different mechanisms. It (1) reduces ischemia-reperfusion-induced inflammatory changes, (2) augments angiogenesis and fibroblast proliferation, (3) promotes neutrophil bacterial killing activity, suppresses clostridial spore and exotoxin generation, and kills anaerobes such as *Clostridium perfringens*, (4) induces vasoconstriction, which in turn reduces vasogenic edema, and (5) may work with growth factors such as plateletderived growth factor (PDGF), which require the presence of oxygen for successful function. As with other medical conditions, it may take up to 30 or more sessions to have good results (9, 19).

In conclusion, considering the limited data, this case report indicates that hyperbaric oxygen therapy is an effective and safe treatment option for sickle cell leg ulcers; however, further studies are needed to confirm this finding.

## Data availability statement

The original contributions presented in the study are included in the article/supplementary material, further inquiries can be directed to the corresponding author.

## Ethics statement

The studies involving human participants were reviewed and approved by Medical Research Council, Hamad Medical Corporation. The patients/participants provided their written informed consent to participate in this study. Written informed consent was obtained from the individual(s) for the publication of any potentially identifiable images or data included in this article.

## Author contributions

All authors listed have made a substantial, direct, and intellectual contribution to the work and approved it for publication.
